# Books and Authors

**Published:** 1908-07

**Authors:** 


**Gonorrhea, its Diagnosis and Treatment.** By **Frederick Baumann,** Ph.D., M.D., Professor of Genitourinary Diseases in the Reliance Medical College, Chicago. 12 mo, pp. 218. Illustrated. New York and London: D. Appleton and Company. 1908. (Cloth, $1.50.)

This, for a small book, is of immense practical value for it very completely covers the entire field of gonorrhea. Written primarily as a guide for the author's students, it was later developed into its present form and placed before the profession. Beginning with the anatomy of the urethra, the pathology of gonorrhea is taken up and fully considered; then diagnosis; the different varieties of gonorrhea, its prognosis, treatments of the various phases of the disorder, and the complications which too frequently appear, even in well conducted cases. The instruments used in the treatment of the disease are described and their use explained. The book is completed by a brief description of the vaccination therapy of gonorrhea. In this the author very properly says, and every worker in the genitourinary field will agree with him, that while it is undoubtedly true that vaccination is of material benefit in some types of gonorrhea, it will never replace the present methods of treatment for the elimination of the gonococcus. The book is of decided value as a reference work.

**Transactions of the American Urological Association.** Sixth annual meeting held at Atlantic City, June 3, and 4, 1907. Edited by **Charles Greene Cumston,** M.D.

This enterprising special association is fortunate in its editor, who has produced a most attractive volume containing excellent material. Though this book is a record of the work done by the association at its sixth annual meeting, it is yet the first time the proceedings have been published in book form. The association was organised through the efforts of Dr. Ramon Guiteras, at Saratoga Springs, June, 1902. This initial meeting was so successful that no longer was there any doubt about the permanency of the association. The sixth session was held at Atlantic City, June 3 and 4, 1907, under the presidency of Dr. Bransford Lewis. The time and the place and the man most appropriately came together. Dr. Lewis chose for the subject of his address 'The Dawn and Development of Urology" in which he set forth most charmingly the status of urology, and the claims of the American Urological Association to recognition and to stability. Dr. Cumston, as editor, has performed his part admirably in the making of a useful volume.

---

**American Practice of Surgery.** A complete System of the Science and Art of Surgery by representative surgeons of the United States and Canada. Edited by **Joseph D. Bryant,** M.D., and **Albert H. Buck,** M.D., New York. Complete in eight volumes. Vol. IV. Imperial octavo, pp. 1010. Profusely illustrated. New York: William Wood & Company. 1907. (Price, cloth, $7.00.)

This volume opens with dislocations, a continuation of the section on diseases and injuries of the joints, from the previous volume. Dislocations always have been an attractive theme for the surgeon and especially for the teacher. Rixford in this dissertation has made it so in an unusual degree. Part fourteen, allotted to the consideration of operative surgery, is contributed to by Charles B. Nancrede, George Ben Johnston, Freeman Allen, F. E. Garland, James F. Mitchell, William L. Rodman, John S. Rodman, Horace J. Whitacre, John M. Keyes, Russell S. Fowler, and James S. Stone.

Part fifteen deals with orthopedic surgery and is contributed to by Charles F. Painter. George D. Stewart, Royal Whitman, and Clarence L. Starr. The strides of orthopedic surgery have been great within recent years and the articles in this section present the subject in its most modern view.

This volume, number four in the series, marks the half-way point in the work which, it will be remembered, is limited to eight volumes. Should the remaining four equal in value and importance the four preceding numbers, and there is every reason to believe they will, it is safe to assert that no work on American surgery has ever given such promise, none has ever reached such a high plane of splendid achievement, as has this treatise of Bryant and Buck.

---

**An Index of Treatment by various writers.** Edited by **Robert Hutchison,** M.D., F.R.C.P., Physician to London Hospital, and **H. Stansfield Collier,** F.R.C.S., Surgeon to St. Mary's Hospital, London. Revised by Warren Coleman, M.D., Professor of Clinical Medicine in Cornell University Medical College. Octavo, pp. 888. New York: William Wood & Co., 1908. (Cloth, $6.00.)

Rarely has it been our good fortune to examine a similar book that contained so much valuable material, so compactly prepared, and so well arranged, as we find in this one. The English editors announce that it is intended to provide the practitioner with a complete guide to treatment in reasonably narrow limits and in convenient form. A reasonably careful examination of the work leads to the conclusion that the purpose of the editors has been met in a most admirable manner. Not only this but all in all, it is a most useful book for the reference library, giving the latest hints as to treatment and including all the newer methods of bacteriotherapeutics. The list of contributors has been well selected and contains the names of many eminent physicians and surgeons,—names familiar on this side of the Atlantic. We bespeak for the work a liberal sale in this country.

---

**Bier's Hyperemic Treatment in Surgery, Medicine and the Specialties.** By **Willie Meyer,** M.D., Professor of Surgery at the New York Post-Graduate Medical School and Hospital. And Professor **Dr. Victor Schmieden,** Assistant to Professor Bier, University of Berlin, Germany. Octavo, pp. 209. Illustrated. Philadelphia and London: W. B. Saunders Co. 1908. (Cloth, $3.00.)

The hyperemic treatment of certain diseases, originally formulated and put into practical employment by August Bier, of Berlin, has attracted attention the world over. One of the authors of this manual, Dr. Willy Meyer, has used the method since it was first published, now some fifteen years ago, and probably has had greater experience with it than any other American surgeon. The other author, Professor Dr. Schmieden, came to this country last year, read papers and traveled extensively lecturing and demonstrating the method, particularly in the eastern portion of the United States. He recognised the need everywhere he went of a comprehensive manual detailing this procedure, hence this volume has been prepared to meet the demand thus made known. The book partakes of the practical character, giving carefully prepared directions for the use of the treatment, all well illustrated by cuts of instruments and pictures of patients undergoing treatment for various affections. While much of the application of the treatment is theoretical there is yet enough accomplished in some diseases to justify its further employment, or to extend it into as yet untried fields. In any event this book should receive the patient study of every physician interested in the method of treatment.

